# Mathematical Modeling of Renal Tubular Glucose Absorption after Glucose Load

**DOI:** 10.1371/journal.pone.0086963

**Published:** 2014-01-29

**Authors:** Andrea De Gaetano, Simona Panunzi, Dimitris Eliopoulos, Thomas Hardy, Geltrude Mingrone

**Affiliations:** 1 CNR-Institute of Systems Analysis and Computer Science (IASI), BioMatLab, Rome, Italy; 2 2nd Department of Internal Medicine, University of Athens Medical School, Hippokration, Greece; 3 Eli Lilly & Co., Indianapolis, Indiana, United States of America; 4 Department of Internal Medicine; Università Cattolica S. Cuore, Rome, Italy; University of Southampton, United Kingdom

## Abstract

A partial differential Progressive Tubular Reabsorption (PTR) model, describing renal tubular glucose reabsorption and urinary glucose excretion following a glucose load perturbation, is proposed and fitted to experimental data from five subjects. For each subject the Glomerular Filtration Rate was estimated and both blood and urine glucose were sampled following an Intra-Venous glucose bolus. The PTR model was compared with a model representing the conventional Renal Threshold Hypothesis (RTH). A delay bladder compartment was introduced in both formulations. For the RTH model, the average threshold for glycosuria varied between 9.90±4.50 mmol/L and 10.63±3.64 mmol/L (mean ± Standard Deviation) under different hypotheses; the corresponding average maximal transport rates varied between 0.48±0.45 mmol/min (86.29±81.22 mg/min) and 0.50±0.42 mmol/min (90.62±76.15 mg/min). For the PTR Model, the average maximal transports rates varied between 0.61±0.52 mmol/min (109.57±93.77 mg/min) and 0.83±0.95 mmol/min (150.13±171.85 mg/min). The time spent by glucose inside the tubules before entering the bladder compartment varied between 1.66±0.73 min and 2.45±1.01 min.

The PTR model proved much better than RTH at fitting observations, by correctly reproducing the delay of variations of glycosuria with respect to the driving glycemia, and by predicting non-zero urinary glucose elimination at low glycemias. This model is useful when studying both transients and steady-state glucose elimination as well as in assessing drug-related changes in renal glucose excretion.

## Introduction

Throughout evolution, higher organisms developed complex and highly specific methods to regulate glucose homeostasis. The liver, pancreas, muscle tissue, gastrointestinal cells and adipocytes interact through neuroendocrine hormones in order to maintain a steady blood glucose concentration and preserve the energy supply to the brain [1].

**Figure 1:**
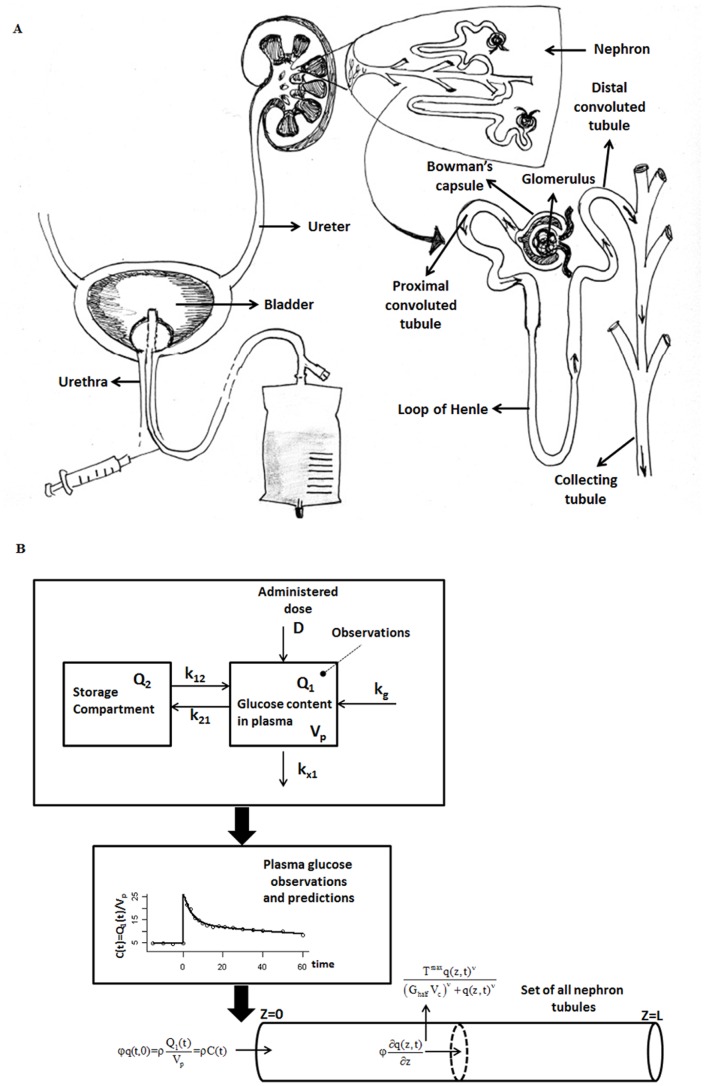
Schematic representation of renal anatomy, experimental set-up, and modeling. Panel A reports a schematic representation of renal anatomy and of the experimental set-up; panel B reports the schematic compartmental diagram of the Glycemia Model as well as of the Tubular System.

Kidneys are an important contributor in the regulation of glycemia (plasmatic glucose levels) [2]. The glomerulus filters approximately 162 grams of glucose per day from plasma, all of which is reabsorbed in tubules under normal conditions [3] (see Figure 1A). In this way, urinary glucose loss is avoided and energy is preserved. Renal tubular cells have the ability to adapt their glucose reabsorption capacity depending on glucose filtration rate, this in turn depending on plasma glucose concentration. Indeed, low-affinity, high capacity sodium glucose cotransporter-2 (SGLT2) and high-affinity, low capacity sodium glucose cotransporter-1 (SGLT1), both located in the proximal tubule of the kidney, increase their activity in presence of increased tubular glucose load [4], [5]. It has been observed that in non-diabetic individuals, with Glomerular Filtration Rate (GFR) between 90 and 120 mL/min per m^2^ Body Surface Area (BSA), essentially complete glucose reabsorptive capacity is maintained up to glucose blood concentrations of about 11 mM [2]. When glycemia exceeds that level, glucose tubular transporters become saturated and urinary glucose excretion increases. The blood glucose concentration at which this phenomenon is observed is commonly known as the Renal Glucose Threshold for excretion (RGT), and the approximately linear above-threshold relationship between hyperglycemia and glycosuria (excretion of glucose into the urine) has been extensively studied, in normal subjects as well as in patients with Type 1 and Type 2 Diabetes Mellitus [6]–[8].

Some published reports, particularly in the early days of the exploration of glucose renal handling, had however advanced critical opinions on the effective existence of a renal threshold mechanism [9]–[12], both because glycosuria was observed at low glycemias, and because variations in glycemia seemed to precede variations in glycosuria. A coherent, if qualitative, explanation of these observations was however not offered, even though the mechanism of tubular reabsorption of glucose was taken for granted at least as early as the work of Richards [13] and Ni and Rehberg [14].

The purpose of the present work is to show that the quick variations in glycemia and glycosuria following a glucose load perturbation are poorly described by assuming a renal glucose threshold, even though the concept of Renal Glucose Threshold is well rooted in common medical and diabetological practice.

We propose here a mathematical model, which incorporates a simple description of the renal tubular glucose reabsorption mechanism. We also discuss the ability of the proposed model to approximate observations better than a model formulation representing the naïve threshold hypothesis.

The model presented here is clearly an oversimplification of renal tubular glucose transport, more complex and detailed models having already been proposed in the literature [15]–[17]. Still, this simple, didascalic model embodies the crucial element, which the naïve Renal Threshold approach lacks, i.e. slow reabsorption. It appears that the simple introduction of this element is sufficient to capture the relevant observed features of delayed glycosuria variations with respect to glycaemia variations and nonzero glycosuria at low glycaemia.

## Materials and Methods

### Ethics Statement

Inpatients from the departments of Internal Medicine of the “Gemelli” hospital, Rome, Italy and “Hippokrateion” General Hospital, Athens, Greece, were enrolled in this study. Patients were considered eligible if they were clinically stable, had a bladder catheter and provided written informed consent for scientific use and publication of the recorded data. The exclusion criteria included history of diabetes and antidiabetic treatment, lack of collaboration during the study and concurrent severe medical illness, such as sepsis.

The protocol was submitted and approved by the Institutional Ethical Committees of the Department of Internal Medicine of Catholic University, Rome and of the “Hippokrateion” General Hospital, Athens and was conducted according to the principles of the Declaration of Helsinki and the Title 45, U.S. Code of Federal Regulations, Part 46, Protection of Human Subjects (2005).

### Subjects and experimental procedures

Five subjects met the criteria and entered the study, Table 1 reports the anthropometric characteristics of the 5 analyzed subjects (3 males and 2 females), along with their computed Glomerular Filtration Rate (GFR).

**Table 1:** Anthropometric characteristics of the studied subjects.

Subject	Gender	Age	Weight (Kg)	Gfr* (ml/min)
1	M	85	72	67.9
2	F	90	50	23
3	M	74	69	14
4	F	91	70	67.5
5	M	88	50	36.1
**mean**		**85.6**	**62.2**	**41.7**
**Standard Deviation**		**6.88**	**11.19**	**25.0**

* **Gfr**: Glomerular Filtration Rate.

All patients were evaluated after overnight fasting, in the supine position. Initially, venous blood samples were obtained for the determination of creatinine, baseline glucose and insulin levels. GFR was then estimated using the Cockcroft-Gault equation [18]. Urine baseline glucose concentration was also measured, using the same quantitative glucose oxidase-based method as for glycemia (Beckman Glucose Analyzer II, Beckman Instruments, Fullerton, Ca, USA). During the study, high urinary flow rate was achieved by continuous infusion of a 500 mL isotonic saline solution (over approximately one hour) and by additional oral intake of 660 mL of liquids.

In order to induce hyperglycemia, 0.33 g/kg body weight of glucose was administered, via intravenous bolus infusion. Blood glucose levels were then promptly measured by fifteen consecutive determinations, with a similar frequency as that employed in the first hour of the standard Intravenous Glucose Tolerance Test [19]. In the meantime, one urine sample of 1 mL was obtained every five minutes from the most proximal part of the Foley catheter inserted in the patient's urethra. Twelve samples were collected; urine glucose concentration was immediately determined on each sample.

## Modelling

### Glycemia model

In order to provide an input function into the glycosuria model (see below) it was necessary to represent plasma glycemia as a time-continuous function. Different approaches were used to represent glucose plasma concentrations following the glucose load: three different models of the time-course of glycemia were tested, differing by type of glucose elimination and by number of compartments. A merely numerical linear interpolation procedure of measured glycemias was also tested in alternative to the three models. The final model chosen for glycemia consisted of a two-compartmental model with linear glucose elimination; this and the interpolation approach were compared with respect to their ability to contribute to the final glycosuria model.

#### The two-compartment glycemia model

The simple two-compartment glycemia model after intravenous (IV) bolus, satisfying the purpose of providing a plausible smooth glycemia input into the glycosuria model, is described by the following two differential equations (the meaning and units of the parameters are reported in Table 2, whereas a schematic representation of the model is reported in figure 1B):
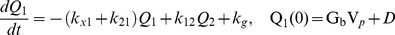
(1)

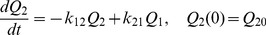
(2)From steady-state conditions, the parameters k_12_ and k_g_ can be determined as follows:
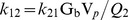
(3)

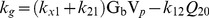
(4)The state variable *Q*_1_ of the above model represents the glucose content in plasma, the variable *Q*_2_ represents glucose in the storage compartment (e.g. interstitial fluid). The parameters *k*_ji_ are the first order transport rates from compartment i to compartment j while *k*_g_ represent the net balance of the constant fraction of hepatic glucose output (HGO) and insulin-independent zero-order (brain) glucose tissue uptake. The initial condition for *Q*_1_ is given by the baseline plasma glucose content (*G*_b_ *V*_p_, where *V*_p_ is the apparent plasma volume and *G*_b_ is the baseline glucose concentration) plus the (instantaneous) intra-venous glucose bolus administered *D*. The storage compartment *Q*_2_ is supposed to have a glucose content equal to *Q*_20_ at equilibrium.

**Table 2:** Model parameter description.

Parameter	Unit of measurements	Description
		**Glycemia model parameters**
Gb	[mM]	basal plasma glucose concentration immediately before glucose administration
V_p_	[L]	glucose distribution volume
D	[mmol]	dose of glucose administered
Q_20_	[mmol]	starting glucose quantity in the storage compartment (interstitial fluid)
k_x1_	[1/min]	glucose elimination rate from plasma compartment
k_21_	[1/min]	glucose transfer rate from plasma compartment to the storage compartment
k_12_	[1/min]	glucose transfer rate from the storage compartment to the plasma compartment
k_g_	[mmol]	net balance of the constant fraction of hepatic glucose output (HGO) and insulin-independent zero-order glucose tissue uptake
		**PTR model parameters**
T^max^	[mmol/(cm min)]	maximal transport rate of glucose from the tubule (maximal glucose reabsorption rate)
G_half_	[mM]	Tubular glucose concentration per cm at which the glucose reabsorption rate is half of its maximum
ν	[#]	Exponent determining the steepness of the reabsorption rate increase with tubular glucose concentration
Vc	[L]	Apparent glucose distribution volume of a single tubule cell
φ	[cm/min]	Velocity with which the filtrate flows along the tubule
W_T_	[min]	Time necessary to completely wash-out one cell
TinTub	[min]	filtrate time in tubule
		**RTH model parameter**
T	[mM]	Theoretical threshold up to which glucose reabsorption takes place
S	[#]	factor multiplying the quantity of glucose excreted in the urine per minute. Typically according to the RTH model it should be equal to 1.
		**Bladder Compartment parameter**
α2	[1/min]	Transfer rate from the bladder to urine
V_B_	[L]	Bladder volume

### The Progressive Tubular Reabsorption (PTR) model of glucose loss

#### Tubular System Description

The model proposed in the present work is a partial differential equation model, which describes the passage of filtrate through one hypothetical tubule (representing the aggregate set of all nephron tubules), where the composition of the filtrate is altered by the selective re-absorption of water and other constituents (such as glucose).

From a physiological point of view the “tubule” represents those parts of the nephrons which have glucose-reabsorbing capacity, mainly the proximal convoluted tubule (see figure 1A).

The variation over time of the cross-section averaged Glucose density *q(t,z)* [mmol/cm] at each position z in the tubule is given by:
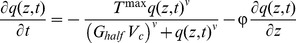
(5)with initial and boundary conditions:
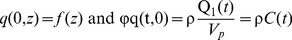
(6)In equation (5) 
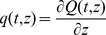
 has dimensions of a spatial density [mmol/cm] and, consequently, 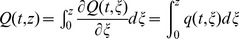, in [mmol], represents the content of glucose within the lumen from position 0 until position z along the tubule. The first term in (5) on the right-hand side represents a saturable re-absorption process, with *T* ^max^ being the maximal transport rate of glucose per cm from the tubule (expressing therefore the maximal glucose reabsorption rate) and with G_half_ being the tubular glucose concentration at which the transport from the tubule is half of its maximum. The steepness of the reabsorption rate, increasing with tubular glucose concentration, is determined by parameter *ν*. The second term represents instead the advective transport of filtrate, with the parameter φ being the velocity with which the filtrate flows along the tubule (assumed for simplicity to have constant length L across all individuals). Notice that the present form of the model assumes diffusion to be negligible.

The parameter *T* ^max^ (mmol/min) is written as
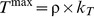
(7)where *ρ* (L/min) is the patient Glomerular Filtration Rate and *k*_T_ is a free model parameter (with units of glucose concentration per cm) to be estimated: this notation has been employed in order to express, for each studied subject, the maximal transport out of the tubule as a function of an apparent, fictitious ‘glucose concentration threshold’ *k*_T_ (the glucose concentration consistent with the maximal transport rate *T* ^max^ if the threshold hypothesis were applicable).

In the boundary conditions (6), *Q*_1_(*t*) [mmol] is the glucose content in plasma as derived by the two compartment model (1)–(2), while *C*(*t*) indicates the glucose plasma concentration [mM].

The glucose content, which reaches the end of the tubule (*φq*(*t,L*)), is transferred into a composite delay compartment representing the collecting tubule, the renal pelvis, the ureter, the bladder and the intraurethral portion of the Foley catheter. This delay compartment is named in the following as the “Bladder Compartment”.

Some crude assumptions have been made in order to represent tubular reabsorption as occurring in a single idealized tubular segment. The cross sectional area of the segment decreases exponentially from what is needed to accommodate a daily flow of ultra-filtrate of approximately 180 L/day to what is needed to produce a daily flow of urine of 2 L/day, assuming constant flow velocity along the tubule. Ionic concentration variations have been neglected, and tubular re-absorptive ability has been assumed to be constant throughout the length of the idealized aggregated tubule. Water reabsorption has been assumed to equal, at each position z, the rate needed to produce the above-mentioned exponential decrease of tubular cross sectional area. In this way a (relative) increase of glucose concentration is produced, effective in driving more distal glucose reabsorption.

Given all of the above assumptions, the main limitation of the model is that it is explicative of a fundamental mechanism rather than being quantitatively appropriate for the determination of the underlying anatomy and physiology. For example, nephron-to-nephron variability of lengths, absorptive and concentrating properties, and positions in the renal pyramids, have all been neglected.

#### Bladder Compartment

Once glucose exits the tubule, it ends up in the composite delay compartment described above, which for clarity is collectively denominated “Bladder”. The description of the variation over time of the glucose content inside the bladder requires the consideration of the urinary flow (*U*_flow_) as well as of the bladder volume (*V*_b_). The urinary flow is approximated by the average urine produced per minute over the total duration of the experiment. The rate of glucose loss (mmol/min) is determined by multiplying *U*_flow_ by the measured glucose concentrations over time. The following equation therefore complete the PTR model:
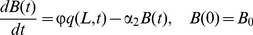
(8)where *B* stands for Bladder glucose content, *L* is the length of the tubular segment and *α*_2_ (min^−1^) is the elimination rate of glucose from the bladder, given by:
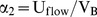
(9)The rate of glucose loss with urine is therefore given by the content of glucose exiting the Bladder compartment per minute, that is:
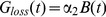
(10)The initial condition of the differential equation (8) for the Bladder follows from the equilibrium condition:
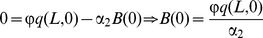
(11)


#### Implementation

The tubule was numerically discretized as a sequence of ten adjacent segments.

At time zero, the system is at equilibrium: the initial condition 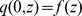 is obtained numerically by having ultrafiltrate (at constant baseline glycemia *G*_b_) pass along the tubule until the glucose content at the very end of the tubule does not vary over time. The quantity of glucose entering the tubule at any given time t, over a time step equal to *Δt*, is 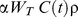 mmol, where C(t) is the current plasma glucose concentration, ρ is the glomerular filtration rate, W_T_ is the time necessary to completely wash-out one segment, that is the time necessary for the filtrate to pass through a single discretization segment of the tubule, and *α* is the ratio between *Δt* and *W_T_* and represents therefore the fraction of the tubular segment travelled by the filtrate over the time interval *Δt*. Notice that the total time in tubules, i.e. the time necessary for ultra-filtrate to exit the tubules as urine, equals the number of discretization segments times *W_T_*_,_ or, which is the same, *L/φ*. The glucose loss rate at time *t* is given by the glucose content in the last discretization segment of the tubule over *W_T_*, i.e.
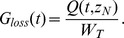
The finite difference method was used to solve the PTR model.

### Renal Threshold Hypothesis (RTH) model of glucose loss

#### The RTH system description

The newly proposed PTR model is here compared with a model representing the conventional Renal Threshold Hypothesis (RTH) proposed by Claude Bernard [20]. The RTH model is described by the following simple system of equations:
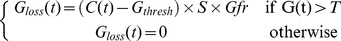
(12)where *C*(*t*) is the plasma glucose concentration at time t, *T* is the threshold glycemia above which urinary glucose loss increases linearly with slope 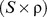, and ρ is the Glomerular Filtration Rate. The dimensionless parameter S should equal exactly 1 according to the threshold theory, because all the glucose exceeding the reabsorption threshold is assumed to be excreted in the urine. In the present work we tested the RTH model once by assigning to S its theoretical value of 1 and once by leaving S free to vary, in case this could contribute to a better data fit.

#### Bladder Compartment

In order to make the two models directly comparable, the same Bladder compartment was also added to the RTH model, which was therefore supplemented with the following differential equation (see eqs. (8) and (10) for explanations):
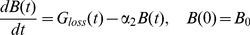
(13)where
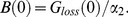
(14)


### A comparison between the two models

Parameter estimates for the two models of urinary glucose loss were obtained using, as the driving continuous plasma glucose concentration time course, both the predicted glycemia over time, derived from the two-compartment model described in the “glycemia model” section, and the linearly interpolated glycemias.

The comparison criteria were based on the visual inspection of the fitting to the observed data and on the loss function value. Parameter estimation was performed by Ordinary Least Squares, minimization of the objective function was achieved by means of a Nelder-Mead Simplex algorithm. Descriptive statistics of quantitative variables as well as estimates of parameters are reported as mean ± standard deviation. All computations were performed in R [21].

## Results

**Figure 2:**
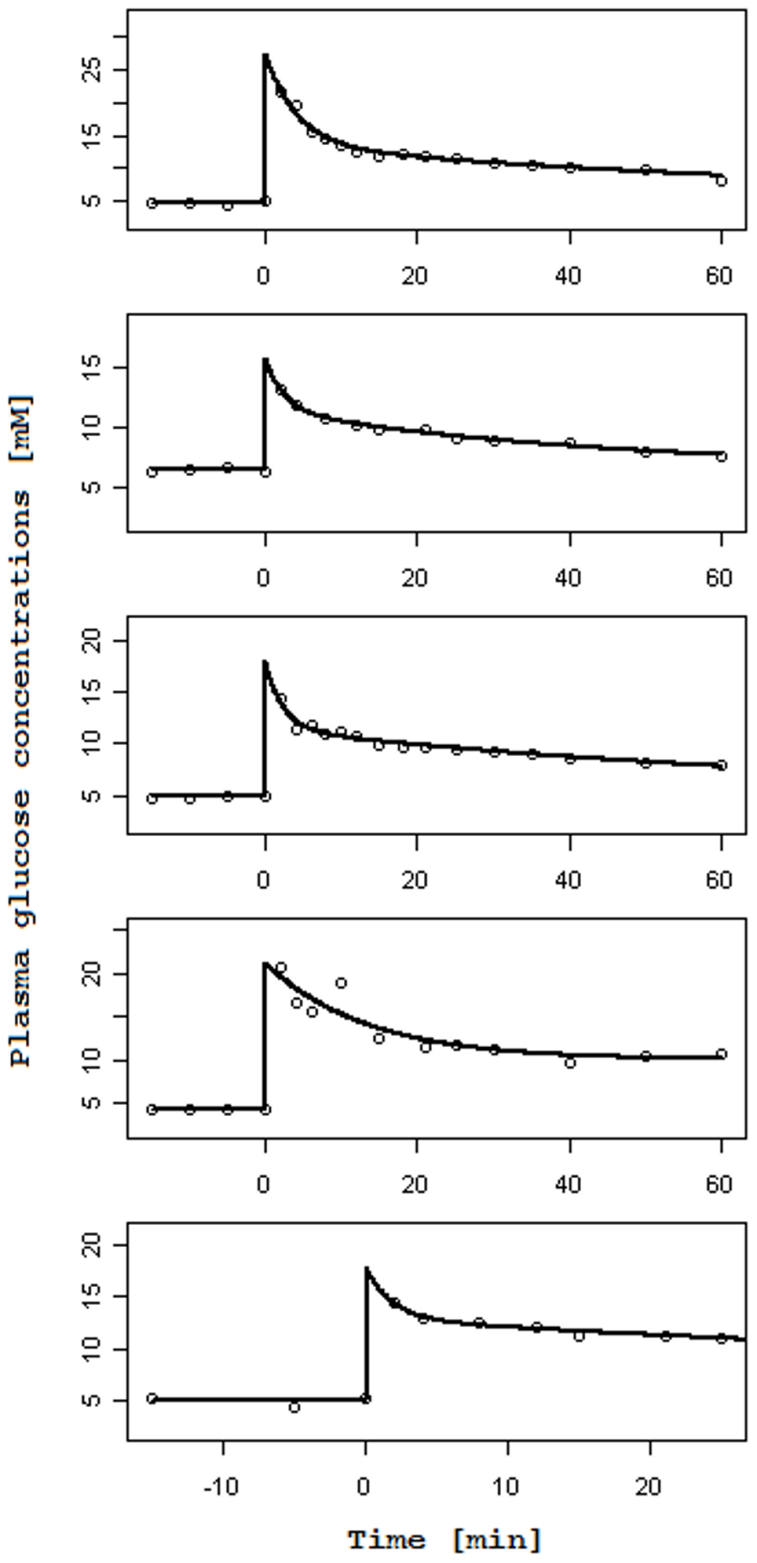
Observed and predicted Plasma Glucose Concentrations. Observed and Predicted Plasma Glucose concentrations (mM) over Time (min) as derived from the two-compartment model for the five analyzed subjects.

Patient average age was 85.5±6.9 years, Glomerular Filtration Rate (ρ) was 41.7±25.0 ml/min. Patients with low GFR were specifically chosen because discrepancies between the traditional RTH and the new PTR model were expected to be large in this class of subjects. Table 3 reports the subject parameter estimates, along with means, standard deviations and coefficients of variation, of the two-compartment model used for the interpretation of observed plasma glucose concentrations after perturbation. The plasma glucose volume *V*_p_ was estimated at 8.05±1.74 L, corresponding to 0.13±0.04 L/Kg, a somewhat smaller value than that estimated for young healthy subjects (0.19 L/Kg) [22]. Figure 2 shows the fits obtained with the chosen two-compartment model for glycemia, for each one of the five studied subjects.

**Table 3:** Parameter estimates for the two-compartment model for glycemia interpretation.

Subject	k_x1_	k_21_	G_b_	Q_20_	V_p_	k_12_	k_g_
1	0.029	0.123	4.62	32.64	5.80	0.10	0.79
2	0.040	0.159	6.39	44.31	9.95	0.23	2.56
3	0.026	0.200	4.87	41.55	9.64	0.23	1.22
4	0.000	0.050	4.35	65.10	7.66	0.03	3.33E-05
5	0.017	0.200	5.02	21.47	7.20	0.34	0.63
**mean**	**0.023**	**0.147**	**5.05**	**41.01**	**8.05**	**0.18**	**1.04**
**Standard Deviation**	**0.015**	**0.063**	**0.79**	**16.15**	**1.74**	**0.12**	**0.96**
**CV%**	**29.81**	**19.18**	**7.02**	**17.61**	**9.66**	**29.59**	**41.20**

Tables 4, 5 and 6 report the parameter estimates obtained with the RTH model (with slope S respectively fixed to 1, Table 4, and free to vary, Table 5) and with the PTR model (Table 6). Each of these tables reports in the left columns the results obtained when glycemias were interpolated and in the right columns the results obtained when glycemias were fitted with the two-compartment model.

**Table 4:** Parameter estimates for the RTH model, Slope fixed to 1.

	Interpolated Glycemia			Fitted Glycemia		
Subject	T	V_B_	Loss	T	V_B_	Loss
1	10.960	0.046	6.03E-03	11.416	0.064	3.28E-03
2	9.029	0.006	6.42E-04	9.227	0.011	8.62E-04
3	6.921	0.013	2.03E-03	7.095	0.024	3.05E-03
4	16.603	0.014	4.81E-04	16.727	0.045	1.63E-03
5	9.636	0.141	3.75E-06	5.018	0.477	1.03E-05
**mean**	**10.630**	**0.044**	**1.84E-03**	**9.897**	**0.124**	**1.76E-03**
**Standard Deviation**	**3.643**	**0.056**	**2.46E-03**	**4.502**	**0.198**	**1.40E-03**
**CV%**	**15.327**	**57.076**	**59.90**	**20.343**	**71.461**	**35.50**

**Table 5:** Parameter estimates for the RTH model, free Slope.

	Interpolated Glycemia				Fitted Glycemia			
Subject	T	S	V_B_	Loss	T	S	V_B_	Loss
1	12.080	1.601	0.073	1.58E-03	11.921	1.174	0.074	2.58E-03
2	10.291	3.478	0.016	2.53E-04	9.971	1.860	0.020	6.84E-04
3	7.920	1.666	0.018	1.14E-03	9.809	5.161	0.090	2.01E-03
4	10.558	0.100	0.010	1.05E-03	13.237	0.225	0.022	1.04E-03
5	10.117	10.000	1.235	3.64E-06	5.018	0.260	0.115	9.96E-06
**mean**	**10.193**	**3.369**	**0.270**	**8.05E-04**	**9.991**	**1.736**	**0.064**	**1.26E-03**
**Standard Deviation**	**1.490**	**3.895**	**0.540**	**6.56E-04**	**3.124**	**2.032**	**0.042**	**1.03E-03**
**CV%**	**6.54**	**51.71**	**89.28**	**36.4**	**13.982**	**52.345**	**29.477**	**36.41**

**Table 6:** Parameter estimation for the PTR model.

			Interpolated Glycemia					Fitted Glycemia						
Subject	T^max^	TinTub	φ*	G_half_	ν	V_B_	Loss	T^max^	TinTub	φ*	G_half_	ν	V_B_	Loss
1	0.870	1.279	1.173	0.590	3.604	0.046	1.81E-03	0.91	1.207	1.243	0.462	5.606	0.068	1.38E-03
2	0.234	1.198	1.252	0.551	4.098	0.004	1.76E-04	0.249	2.072	0.724	0.198	18.805	0.010	1.30E-04
3	0.136	2.721	0.551	0.671	2.089	0.009	1.52E-04	0.146	3.520	0.426	0.659	2.077	0.019	5.61E-05
4	1.392	1.000	1.500	1.248	1.272	0.010	4.32E-06	2.459	1.981	0.757	5.334	0.774	0.014	1.32E-04
5	0.411	2.127	0.705	0.001	0.534	0.154	4.89E-08	0.405	3.464	0.433	0.156	13.089	0.272	2.96E-07
**mean**	**0.609**	**1.665**	**1.036**	**0.612**	**2.319**	**0.045**	**4.28E-04**	**0.834**	**2.449**	**0.717**	**1.362**	**8.070**	**0.077**	**3.39E-04**
**Standard Deviation**	**0.521**	**0.731**	**0.395**	**0.443**	**1.513**	**0.064**	**7.76E-04**	**0.955**	**1.010**	**0.333**	**2.230**	**7.676**	**0.112**	**5.82E-04**
**CV%**	**38.273**	**19.639**	**17.060**	**32.374**	**29.164**	**63.722**	**81.07**	**51.19**	**18.445**	**20.776**	**73.236**	**42.536**	**65.201**	**76.81**

* We consider a reabsorptive length of approximately 50% of a standard human nephron (3 cm).

The average threshold for glycosuria, as derived from fitting the RTH model, varied between 9.90±4.50 mmol/L and 10.63±3.64 mmol/L, depending on whether fitted or interpolated glycemias were used and whether the slope coefficient S was kept fixed to the theoretical value of 1 or was left free to vary. The corresponding average maximal transport rates (given by the product of threshold glycemia and Glomerular Filtration Rate) varied between 0.48±0.45 mmol/min (86.29±81.22 mg/min) and 0.50±0.42 mmol/min (90.62±76.15 mg/min). The volumes estimated for the delay “bladder” compartment varied between 0.044±0.056 L and 0.27±0.54 L.

When the slope coefficient was left free to vary, its value oscillated widely (3.37±3.89 and 1.74±2.03 with interpolated and fitted glycemias respectively).

The average maximal transports rates, as derived from fitting the PTR Model, varied between 0.61±0.52 mmol/min (109.57±93.77 mg/min) and 0.83±0.95 mmol/min (150.13±171.85 mg/min) depending on whether interpolated or fitted glycemias were used. The volumes estimated for the delay “bladder” compartment varied between 0.04±0.06 L and 0.08±0.11 L. The time spent by glucose inside the tubule before entering the delay bladder compartment was estimated at 1.66±0.73 min for the interpolated case and at 2.45±1.01 min for the fitted case.

**Figure 3:**
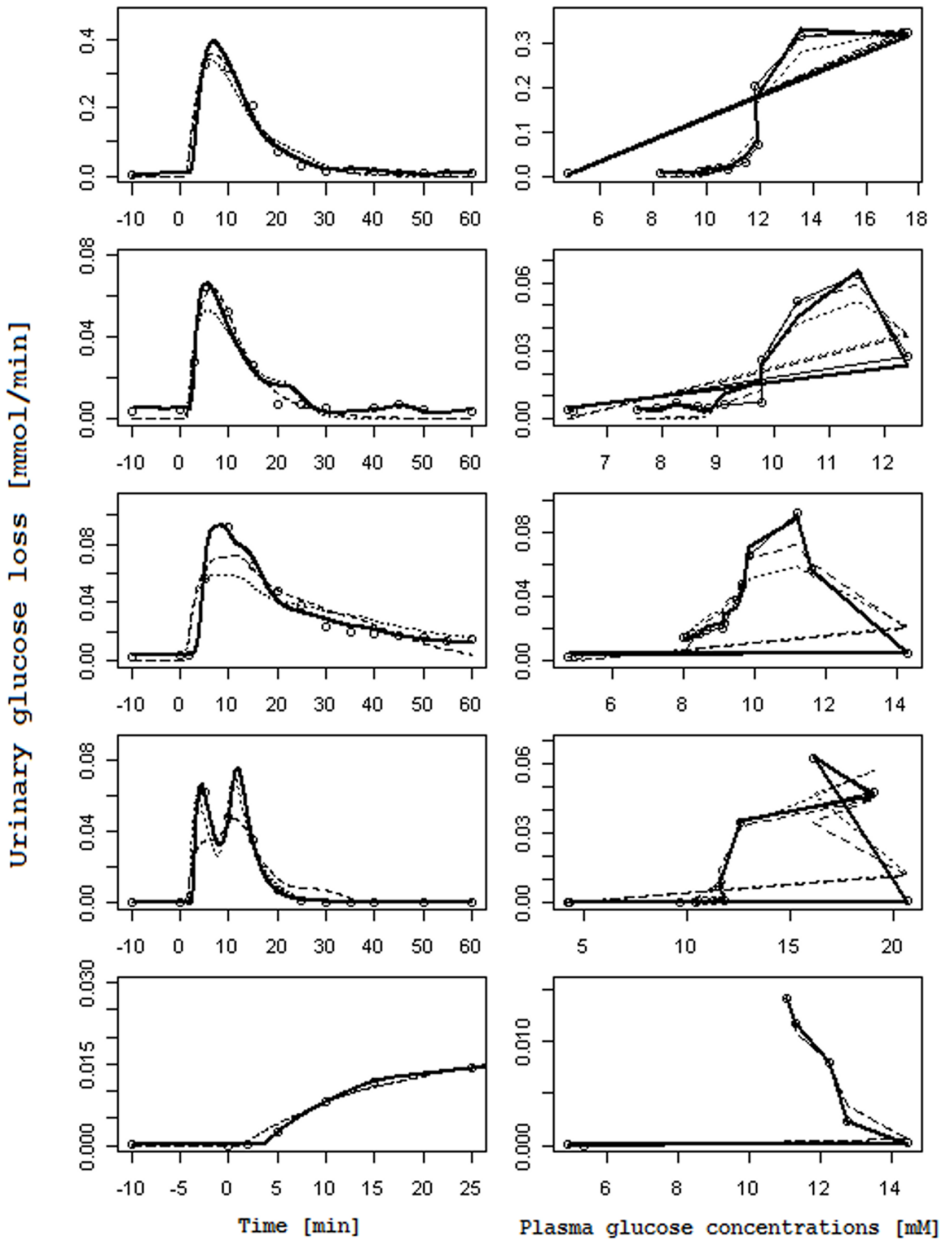
Observed and predicted urinary glucose loss when plasma glucose concentrations are interpolated. Columns on the left report the observed Urinary glucose loss [mmol/min] (circles) over Time [min] along with their prediction obtained with the RTH model letting the Slope vary (dashed line), with the RTH model fixing the Slope at 1 (dotted line) and with the PTR model (thick continuous line). Columns on the right report the same observed Urinary glucose loss (circles), linked with a thin continuous line, in correspondence of the interpolated observed Plasma glucose concentrations [mM], along with urinary glucose loss predictions obtained with the RTH model letting the Slope vary (dashed lines), with the RTH model fixing the Slope at 1 (dotted line) and with the PTR Model (thick continuous line).

**Figure 4:**
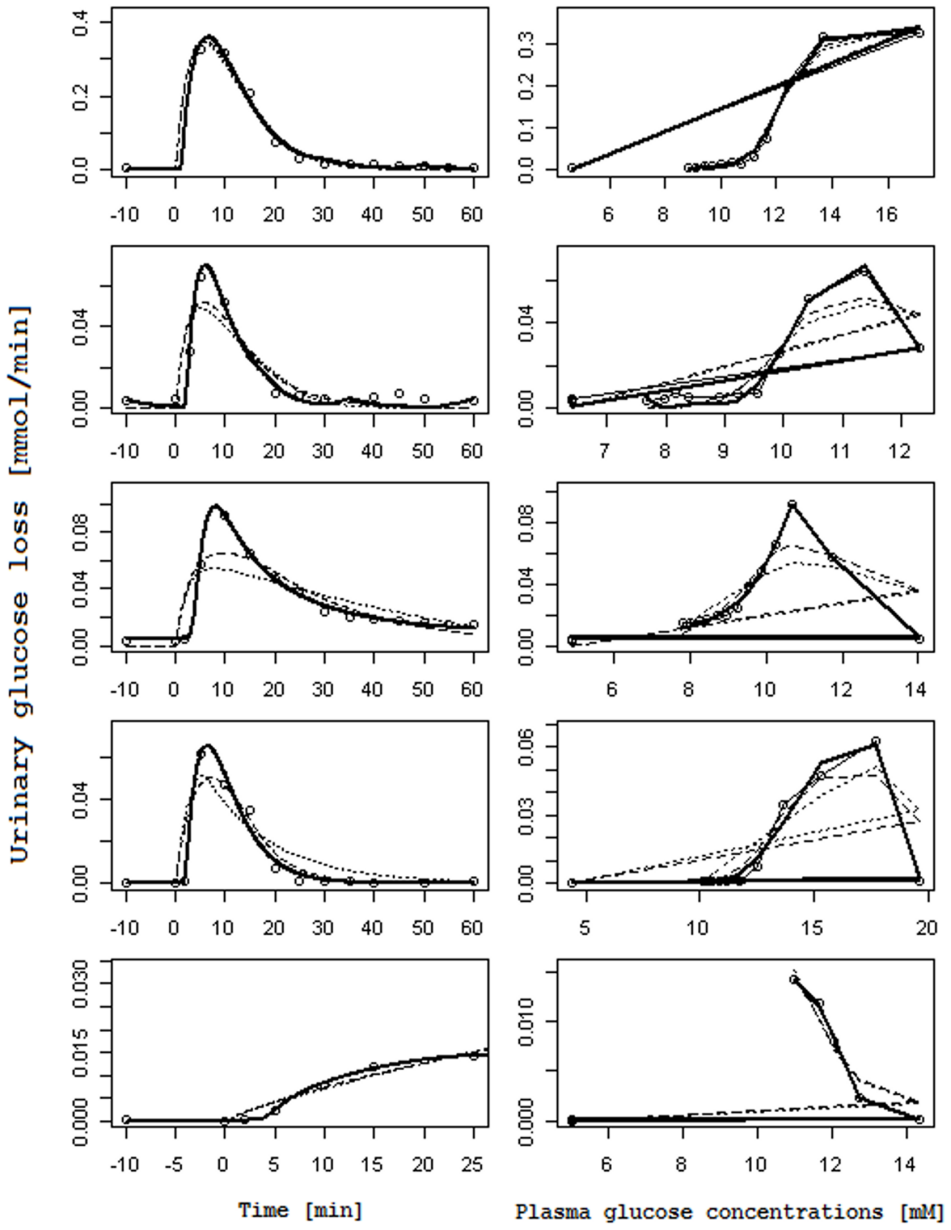
Observed and predicted urinary glucose loss when plasma glucose concentrations are fitted with the two-compartment model. Columns on the left report the observed Urinary glucose loss [mmol/min] (circles) over Time [min] along with their prediction obtained with the RTH model letting the Slope vary (dashed line), with the RTH model fixing the Slope at 1 (dotted line) and with the PTR model (thick continuous lines). Columns on the right report the same observed Urinary glucose loss (circles), linked with a thin continuous line, in correspondence of the predicted Plasma glucose concentrations [mM] s derived from the two-compartment model for glycemia, along with urinary glucose loss predictions obtained with the RTH model letting the Slope vary (dashed lines), with the RTH model fixing the Slope at 1 (dotted line) and with the PTR Model (thick continuous line).

Figures 3 and 4 report, for each subject, the observed glucose loss in the urine along with their predictions: Figure 3 shows the fits obtained when using interpolated gycemias, Figure 4 when using model-predicted glycemia as input function; on the left columns glucose loss over time is reported, whereas on the right columns urinary glucose loss is plotted against the corresponding glucose plasma concentration (showing therefore trajectories in phase space). In the phase space plots successive observations are linked with line segments: the first observation lies typically to the low left (low glycemia, low glycosuria at basal) and as time goes on the subject's state moves along a generally counter-clockwise trajectory or loop, moving to the low right first (i.e. increment of glycemia without much increment in glycosuria), then moving up or upwards and leftwards (i.e. increasing glycosuria with stable or decreasing glycemia), and ending up at the low left again (i.e decreasing glycosuria and glycemia towards basal values); the amplitude of the loop is related to the delay with which variations in glycosuria follow variations in glycemia.

Comparison between the two models shows that the PTR model performs better than the RTH model both when using interpolated plasma glucose concentrations and when using fitted values: the loss function for the PTR model is roughly 53% of the Loss function obtained with the RTH model for the interpolated case and 27% for the fitted case. The better performance of the PTR model can be appreciated from visual inspection of Figures 3 and 4: the PTR model is able to generate a wide loop, adhering to the actual observations by correctly reproducing the delay of variation of glycosuria with respect to the driving glycemia, and to predict low observed urinary glucose concentrations, which the threshold model predicts at zero (glucose loss occurring only when plasma glucose concentrations are above the threshold).

It is of some interest to study qualitatively, from the structure of the more mechanistic Progressive Tubular Reabsorption model, those conditions, if any, under which the Renal Threshold model may satisfactorily approximate it. To this end, a non-dimensionalization of the PTR model is presented in the Appendix S1, where the conclusion is drawn that the Threshold model actually approximates the PTR model's behavior only in the two extreme cases of zero or complete tubular absorption of glucose: in intermediate situations (namely, where tubular reabsorption exists but may not be complete) the predictions of the threshold model substantially diverge from those of the PTR.

## Discussion

The importance of understanding the mechanisms through which the human body maintains the equilibrium and the correct function of its organs (homeostasis) was emphasized from the beginning of the nineteenth century. Sugar metabolism was in fact one of the topics to which much attention was paid. It was appreciated that the kidneys help to maintain a constant plasma glucose concentration by excreting the excess in the urine. The first studies, carried out by Claude Bernard [20] (who coined the *milieu interieur* term at the basis of the concept of homeostasis), led to the conclusion that glucose is not excreted in the urine until it exceeds a certain concentration in plasma. The concept of the “blood sugar threshold” was so well established in the medical common wisdom that, on this basis, subsequent studies focused on determining the numerical value of this threshold [23], [24]. Faber and Norgaard [25], [26] found that the threshold glycemia could vary between different individuals, but that it tended to remain constant during each individual's entire life. Faber [11] found that the glycemic threshold could be so low as to give rise to glycosuria for almost any level of glycemia. He performed a series of experiments after alimentary ingestion in order to assess the threshold level characteristic of each individual. On the same line, a later work by Himsworth [12], supported the concept that urinary sugar content is proportional to the excess blood sugar concentration over the renal threshold, producing evidence that the amount of reabsorbed sugar is determined by the sugar concentration in peritubular capillaries. Thereafter many papers, published since the early twentieth century until today, have accepted the renal threshold hypothesis, at least as a convenient shortcut. Even very recent work [3], aimed at emphasizing the role of the kidneys in glucose homeostasis and at highlighting the possibility of treatment of diabetes through SGLT2 inhibitors, embraces the idea that glucose reabsorption by the proximal tubule increases linearly with increasing glucose concentration up to a theoretical threshold (approximately equal to 11 mM), and that above this concentration the reabsorption system becomes saturated and all the filtered glucose exceeding the threshold is excreted in the urine. The only moderating considerations, with respect to this all-or-none response, concern the ‘splay’, i.e. the softening of the sudden transition between no secretion and linear elimination, due to the concurrent action of a population of nephrons with similar, but different, thresholds [3].

Hypothesizing the actual existence of a fixed threshold in renal glucose re-absorptive ability may however appear rather artificial. This hypothesis had in fact been criticized as early as 1918 by Benedict et al. [10], who also observed that glycosuria was in fact present at low glycemias. Besides the general idea that *natura non facit saltum* [27], particularly in physiology, two features immediately visible from early published glycemia-glycosuria datasets [11], [12] are inconsistent with the naïve threshold hypothesis: the presence of (a small degree of) glycosuria at very low glycemias (confirming Benedict's original observation); and the delay of the variations in glycosuria with respect to the corresponding variations in glycemia, again confirming previous observations by Frank [9]. While the second phenomenon could be explained, at least in part, by post-renal-excretion mechanisms (e.g. by a delay compartment such as the bladder), the first seems outright incompatible with the RTH: neither can be explained in terms of the nephron population splay.

The present work proceeds from the consideration that a model of glomerular glucose filtration and consequent progressive tubular glucose reabsorption would be compatible with the above-mentioned observations. We reasoned that discrepancies between the RTH model and such a Progressive Tubular Reabsorption (PTR) model would have been largest when considering low rates of flow through the tubule (i.e. low Glomerular Filtration Rates), rapid variations in glycemia, and frequent precise sampling of urinary glucose concentrations. For this reason we designed a hyperglycemic perturbation experiment (based on an IV glucose bolus) in elderly subjects with naturally low GFR's and already in-dwelling Foley catheters (allowing us to frequently sample urine near the urethers).

A simple *Gedankenexperiment* highlights the different results which would be obtainable with the RTH and PTR models: suppose we were able to acutely raise glycemia to a very high level, keep it at that level for a few seconds, then drop it suddenly to normal again. In this case the RTH model would predict glycosuria to occur (since the renal threshold is substantially exceeded), while the PTR model would predict no or little glycosuria to occur (since the total glucose dose delivered to the tubule would be small, given the short time interval of the excess, and the tubule would be able to reabsorb it).

The aim of the present work is therefore that of describing a mathematical model, as simple as possible (but not simpler than that), to represent the way in which nephrons reabsorb filtered glucose and determine the extent of glycosuria after a glucose load. The proposed PTR model is a partial differential equation (PDE) model, according to which filtered glucose, passing through the renal tubule, is progressively reabsorbed by means of a saturable mechanism; the non-reabsorbed glucose is then excreted in the urine through a “storage” bladder compartment.

In order to fit predicted to observed glycosuria rates, both the PTR model and the comparison RTH model need continuous glycemia as input (“forcing”) function. Two possible ways to reconstruct driving glycemia from observations are linear interpolation of observations and modeling of glycemia itself. The use of interpolated noisy experimental observations for the driving signal (glycemia in this case) gives rise to statistical parameter estimation pitfalls (see Panunzi et al. [28] for a thorough discussion of this issue in a different physiological context), leading to an essentially incorrect representation of the phenomenon of interest (glycosuria in this case) and flawed parameter estimates for the model. However, both interpolation and modeling of driving glycemia were studied in the present work, in order to leave no doubts as to the fact that the results obtained do not depend on the specific glycemia model assumed. Looking at the results, it can be appreciated that the modeling approach yields lower coefficient of variation of the parameters and qualitatively more believable time-courses in both glycemia and glycosuria.

As originally hypothesized, the results highlight two main differences between the PTR and RTH models. The first is the ability of the new formulation to predict non-zero glycosuria even when plasma glucose concentrations are low (as determined by careful measurements in specifically conducted studies). All the analyzed subjects of the present series were in fact measured for glucose loss at basal glycemia (on average two measurements per patient in the 10 minutes preceding the glucose bolus) and at the end of the 60-minute post-injection observation period: for all subjects a measurable quantity of glucose in the urine was observed in correspondence of low plasma glucose concentrations. While the RTH model is unable to predict glucose loss occurring below the theoretical threshold, the proposed PTR model allows for (moderate) glycosuria at low glycemias through the hypothesized nonlinearity of the tubular glucose excretion efficiency (Eq. 1). The second feature, which is prominently displayed by the PTR model, is the necessary delay with which variations of glycosuria follow variations in the driving glycemia. This phenomenon can be best appreciated looking at the right columns of the Figures 3 and 4, and is particularly evident for subjects 2, 3 and 4. In the phase-space plots, the hysteresis of the glycemia-glycosuria system gives rise to trajectories, which are far from the theoretical straight line (travelled from lower left to upper right for increasing and in the opposite direction for decreasing glycemias), which would be predicted by the RTH (according to which all the glucose exceeding the threshold is instantaneously excreted in the urine). The addition of a bladder compartment does allow the RTH model to also show a degree of hysteresis, but the actual observations are much more closely approximated by the PTR model: the delay with which variations in ultra-filtrate glucose concentrations translate into urinary glucose concentrations (the transit time in tubules is estimated between about 1.7 to 2.5 minutes) determines the loop in phase space from low left to low right then back to starting position.

It is interesting to note that the numerical parameter estimates from the PTR model are in good agreement with already published results. The maximal transport rate for glucose has been reported [3] as approximately 375 mg/min on average for healthy subjects. The corresponding parameter estimate from the PTR model indicates an average value of about 150 mg/min in the studied sample, a value lower than half of that reported by De Fronzo et al. [3], but still very reasonable considering the somewhat compromised renal excretion of the observed patients (the glomerular filtration rate being on average 42.5 ml/min instead of the 125 ml/min of healthy subjects [3]). On the other hand, the maximal transport rate estimated from the fitted threshold model is about 80 mg/min, that is 20% of the reported value for healthy subjects, all the while with a glycemic threshold of about 10 mM, a very comparable value to that estimated by De Fronzo et al. (11 mM).

It must be underscored that the original Renal Threshold Hypothesis assumes that all filtered glucose above the threshold is actually lost in the urine. In other words, with reference to Eq. 2, the value of the coefficient S according to the original RTH is exactly 1. In fact, it makes very little physiological sense to allow S to take values greater than 1 (which would be equivalent to hypothesizing that, above threshold, more glucose is lost in the urine than what exceeds the threshold itself), while values below 1 would be theoretically possible (sub-total loss). Again in order to clear the field from any possible residual doubts, we fitted observations with the RTH model both fixing S to its theoretical value of 1 and leaving it free to vary. While a modest improvement in the fit to observations is appreciable using free S parameter, its average estimate is 1.7 (modeled glycemia) or even 3.4 (interpolated glycemia), which is not very credible, and the remaining parameters do not change materially (e.g. maximal transport goes from 86.3 mg/min with S fixed to 1 to 81.03 mg/min with S free, modeled glycemias, with similar threshold glycemias, 9.90 mM vs 10.0 mM).

Some confusion may stem from the definitions used. If, instead of loosely referring to the relationship between glycemia and glycosuria in general, we were to characterize specifically the relationship between steady-state glycosuria and steady-state glycemia (after the prolonged maintenance of an hypothetically constant level of glycemia), we would find, even with the PTR model, a nearly perfect Renal Threshold phenomenon, i.e. glycosuria would be minimal (but not zero), constant up to a given threshold, and would then rise linearly (with slope S = 1) as successive steady state situation are achieved, at progressively increasing above-threshold glycemias. If the experimental set-up were such that average glycosuria is monitored every, say, half hour, the results would support *prima facie* the RTH. When however frequent sampling is used, such that the transient adaptation of glycosuria to glycemia via the tubular reabsorption mechanism can be observed, then the relative asynchronous variations of glycemia and glycosuria can be clearly appreciated. If in addition precise urinary glucose concentration measurements are obtained, the simplifying tenet of zero glycosuria below threshold must be abandoned.

It is of historical interest to observe that precise clinical measurements have in fact been available since 1918 [10]–[12] and that they have been subsequently neglected in favor of the more readily employed RTH approximation.

Beyond the present ‘proof-of-concept’ study, the PTR model could be utilized in glucose metabolism research where an accurate assessment of the degree of glycosuria would be desirable, since it more accurately predicts urinary glucose loss across a wide range of glycemias (both above and below accepted ‘thresholds’) and is not dependent on steady state conditions. In particular, the PTR model could be utilized to reevaluate questions regarding the effects of SGLT2 inhibitors, such as predicting the decrease in efficacy of these agents as GFR declines.

## Conclusions

The Renal Threshold Hypothesis is a simplifying interpretation of the actual behavior of glucose reabsorption in the nephron as glycemia rises; it is reasonable in case of attainment of near-equilibrium, for constant or slowly-varying glycemia.

A better model, based on simple PDE dynamics, exploits the progressive reabsorption of glucose along the tubule to yield renal glucose excretion rates close to actual observations, both during rapid transients and near Steady State, and is able to reproduce observed glycosuria at low glycemias.

## Supporting Information

Appendix S1 **Non-dimensionalization of the Progressive Tubular Reabsorption (PTR) model.**
(DOCX)
