# Supplementary material for: Mathematical Modeling of Renal Tubular Glucose Absorption after Glucose Load
Source: PLoS One. 2014 Jan 29;9(1):e86963. doi: 10.1371/journal.pone.0086963 (PMC3906102; doi:10.1371/journal.pone.0086963)
Supplement: Appendix S1 — Non-dimensionalization of the Progressive Tubular Reabsorption (PTR) model. (DOCX) [file pone.0086963.s001.docx]

**Appendix S**

***Non-dimensionalization of the Progressive Tubular Reabsorption (PTR) model***

Let $q=q(t,z)=\frac{\partial Q(t,z)}{\partial z}$, with q [mmol/cm] being the spatial density of glucose (quantity of glucose per cm of tubule), and consider the following boundary value problem:

, with boundary conditions:

,

where *C(t)* [mmol/L] is the concentration profile over time at the origin of the tubule (i.e. the concentration profile of glucose in plasma), *ϕ* [cm/min] is urine flow velocity, and ρ is the glomerular filtration rate (tipically 0.125 L/min in the healthy adult).

From the above formulation, the solution *q(t,z)* can be formally derived.

The model can also be simplified by performing a change into the coordinates of a moving frame. Setting

$\xi=z$ and , we can write

$$\frac{\partial q(t,z)}{\partial t}=\frac{\partial q(\tau,\xi)}{\partial\tau}\frac{\partial\tau}{\partial t}=\frac{\partial q(\tau,\xi)}{\partial\tau}$$

which implies:

and therefore

, that is:

,

where *f(q(τ,ξ))* is the nonlinear reabsorption function:

$f(q(\tau,\xi))=\frac{q(\tau,\xi)^{\nu}}{\left(G_{half}V_{c}\right)^{\nu}+q(\tau,\xi)^{\nu}}$.

From the above simplification we are able to find at least a numerical approximation of *q(τ,ξ)*, for every *τ*, over the domain of *ξ*, $\tilde{q}_{\tau}(\xi)$. Hence , or allowing us to (numerically) compute a glucose density profile over the tubule at any time t,

.

In order to make the system non-dimensional, set

$Q(0,L)=\int_{0}^{L} q(0,\theta)d\theta$ and $q^{,}=q\frac{L}{Q(0,L)}=\frac{q}{\bar{q}_{0}}$ where $\bar{q}_{0}=\frac{Q(0,L)}{L}$.

It follows that

.

Expressing our model in this way allows us to consider the behavior of the system with regards to the constant *Da* representing the non-dimensional reabsorption parameter (Damköhler number) with .

If *Da* << 1(weak reabsorption), then . If *Da* >> 1(strong reabsorption), then $\tilde{G}_{loss}(t)\simeq0$.

In other words, for very small *Da* the lack of reabsorption implies a very low “Renal threshold”, with the consistent behavior of both PTR and Renal Threshold models predicting that urinary glucose output at any given time *t* equals exactly the amount of glucose entering the tubule at the time L/ϕ (taking into account the time necessary for urine to flow through the whole tubular length). Conversely, for very large *Da* both models would predict an “infinite” threshold, with reabsorption of essentially all the ultra-filtered glucose. For intermediate values of *Da* the predictions of the two models would diverge.
